# Searching for effective strategies to reach boys and young men; a mixed-methods study protocol for youth clinics in Sweden

**DOI:** 10.1186/s12913-024-11673-x

**Published:** 2024-10-03

**Authors:** Mazen Baroudi

**Affiliations:** Department of Epidemiology and Global Health, Umeå University, Umeå, Sweden

**Keywords:** Sexual and reproductive health, Adolescent boys, Young men, Demand, Youth clinics, Intervention

## Abstract

**Background:**

Efforts to engage boys and young men in sexual and reproductive health (SRH) services in Sweden remain limited, with only a small proportion accessing youth clinics, the primary providers of such services. Existing initiatives are often ad-hoc and lack institutionalization within public policy and practice. This study aims to identify feasible and effective interventions to improve boys’ and young men’s access to youth clinics in Sweden.

**Methods:**

Employing a mixed-methods approach, this study investigates interventions, strategies, and factors influencing access to SRH services for boys and young men in Sweden. Firstly, a systematic literature review will be conducted to identify evaluated interventions globally. Secondly, strategies to attracts boys and young men in youth clinics in Sweden will be mapped. Thirdly, case studies in two regions in Sweden – chosen for their demographic and geographic diversity – will be conducted interviewing healthcare providers, managers, policymakers, and boys and young men. Lastly, Q-methodology will be used to rank all identified strategies. Healthcare providers and managers will rank these strategies based on their perceived effectiveness and feasibility while boys and young men will rank the interventions based on perceived effectiveness.

**Discussion:**

The added value of this project is generating robust evidence regarding boys and young men’s involvement in SRH services, especially their access to youth clinics. This is crucial for (1) developing gender-sensitive services and service delivery models that effectively promote young men’s SRH; (2) informing future young men’s health policies ensuring that their unique SRH concerns are addressed; and (3) improving young men’s participation in SRH provision. This will ultimately foster a culture of shared responsibilities and advance gender equality.

## Background

Sexual and reproductive health (SRH) is an essential part of human rights safeguarded by international laws and agreements. The realization of SRH necessitates the respect, protection, and fulfillment of the sexual and reproductive rights of all individuals [1, 2]. Upholding men’s rights to SRH holds significance for multiple reasons. Engaging more boys and young men can lead to a reduction in unsafe sexual practices, sexually transmitted infections (STIs), HIV infections, and unwanted pregnancies [3]. Consequently, this fosters sexual health and development, ultimately aiming to enhance overall quality of life [3, 4]. Additionally, SRH services provide a clinical avenue for addressing men’s broader health needs, a growing area of focus, particularly considering global statistics indicating that men have shorter life expectancy than women and are disproportionately represented in almost all main burden-of-disease category [5]. Increasing the participation of boys and young men in youth clinics could serve as a gateway to accessing other services provided therein, such as mental health services, which are also underutilized by boys and young men [6]. Moreover, involving men in discussions surrounding SRH plays a crucial role in promoting and achieving an equitable share of responsibilities for reproductive health. This involvement challenges harmful gender norms and attitudes contributing to gender equality and fostering a more equitable enjoyment of sexual and reproductive rights for both women and men [3, 7, 8].

Research on young men’s access to SRH services is limited. Our recently published scoping review revealed a dearth of literature concerning boys and young men and SRH services in the Nordic countries. The review identified social and gender norms, along with healthcare providers’ knowledge and attitudes, as crucial barriers to men’s access to SRH services [9, 10]. Only one study, conducted in Norway, explored boys’ and young men’s access to youth clinics, highlighting low awareness about the clinics and perceiving them as primarily serving women [11]. Similar perceptions of youth clinics in Sweden were also reported by the Swedish youth clinics’ association (FSUM) [6].

Hegemonic masculinity and prevailing social and gender norms often serve as explanations for the involvement of boys and young men in risky sexual behaviours, hypersexuality, heteronormativity, and a reluctance to seek help [6, 11–14]. However, relying solely on this explanation may hinder the exploration of structural and healthcare factors that can contribute to or exacerbate these behaviours. For instance, a study examining healthcare providers’ (HCPs) attitudes toward men and masculinity in Sweden revealed that HCPs’ attitudes may inadvertently exclude men, highlighting the need for interventions to address organizational barriers and improve men’s access to sexual and reproductive health (SRH) services [10]. Furthermore, the predominant focus of SRH programs and services on women may neglect men’s SRH needs [9, 15]. A national survey on sexual and reproductive health and rights conducted in Sweden in 2017 indicated that over half of men seeking SRH services for sexual function-related issues felt they did not receive adequate assistance, suggesting unmet SRH needs [16].

Our pilot study for this project interviewing HCPs working in youth clinics in Sweden identified a range of individual and organizational factors influencing the utilization of boys and young men of these facilities [17]. Participants highlighted boys’ and young men’s perception of youth clinics as exclusively for girls or for STI testing, which acts as a barrier to seeking care, underscoring the impact of gender norms on health-seeking behaviours [17]. Moreover, participants called for organizational changes within youth clinics to foster inclusivity and equality in care provision. Their suggestions included dedicated receptions for young men, increased hiring of male HCPs, flexible opening hours, reduced waiting times, and implementation of digital solutions [17].

Although youth clinics in Sweden are highly perceived as youth friendly [18, 19], Only 10 to 15% of visitors to youth clinics in Sweden are boys and young men [6]. The Swedish Society for Youth Centers (FSUM) has noted a lack of staff knowledge regarding the SRH needs of boys and young men, highlighting a pressing need to enhance youth clinics’ capacity to address these needs [6]. Efforts to involve boys and young men in SRH in Sweden remain limited to small-scale and short-term initiatives that are not integrated into policy and practice [20]. For instance, many youth clinics have implemented ad-hoc strategies aimed at increasing access for boys and young men, but these interventions are generally isolated, and there is a lack of reports or research on their impact [6].

Given that youth clinics are the primary providers of SRH services for young people in Sweden, this project will focus on enhancing boys’ and young men’s access to these clinics. Through mixed-method research, the aim of this study is to identify the most feasible and effective interventions/strategies to attract boys and young men to youth clinics, addressing the following questions:

1. What interventions to increase boys and young men’s access to SRH services has been evaluated globally?
2. What strategies are used to attract boys and young men to youth clinics in Sweden?
3. What factors hinder or facilitate boys and young men’s access to youth clinics in Sweden?
4. Which strategies are more effective and feasible to improve the access of boys and young men to youth clinics in Sweden?

## Method/design

### Study setting

Youth clinics in Sweden serve as primary healthcare facilities designed for individuals aged up to 22–30 years (with the upper age limit varying by clinic) [6, 21]. Youth clinics serve as primary locations for obtaining information about SRH and relationships, undergoing testing for STIs/HIV for young people, and seeking advice regarding abortion services in addition to mental health consultations and other services. Although girls and young women frequently visit youth clinics, supplementing their SRH knowledge acquired in schools, boys and young men are not reached to the same degree [22].

There are approximately 250 youth clinics situated throughout the country. Visiting these clinics is free of charge, and all youth, including asylum seekers and undocumented immigrants, are entitled to access these services. Nonetheless, existing literature indicates disparities in the utilization of these services among diverse youth populations, such as males and migrants [19, 21].

### Study design and theoretical framework

The study will employ the ecological framework of access [23]. This framework conceptualizes access as a process with various stages before and after the utilization of youth clinics. Recognizing access as a cyclical process, it acknowledges that past experiences with youth clinics influence future utilization. While examining all five stages of access, the project will particularly emphasize approachability, which refers to “the degree to which services can be obtained”. This involves ensuring that users are aware of youth clinics and perceive them as beneficial, that healthcare providers possess sufficient knowledge about boys’ and young men’s SRH, and that youth clinics implement effective strategies and outreach activities to attract boys and young men [23].

**Fig. 1:**
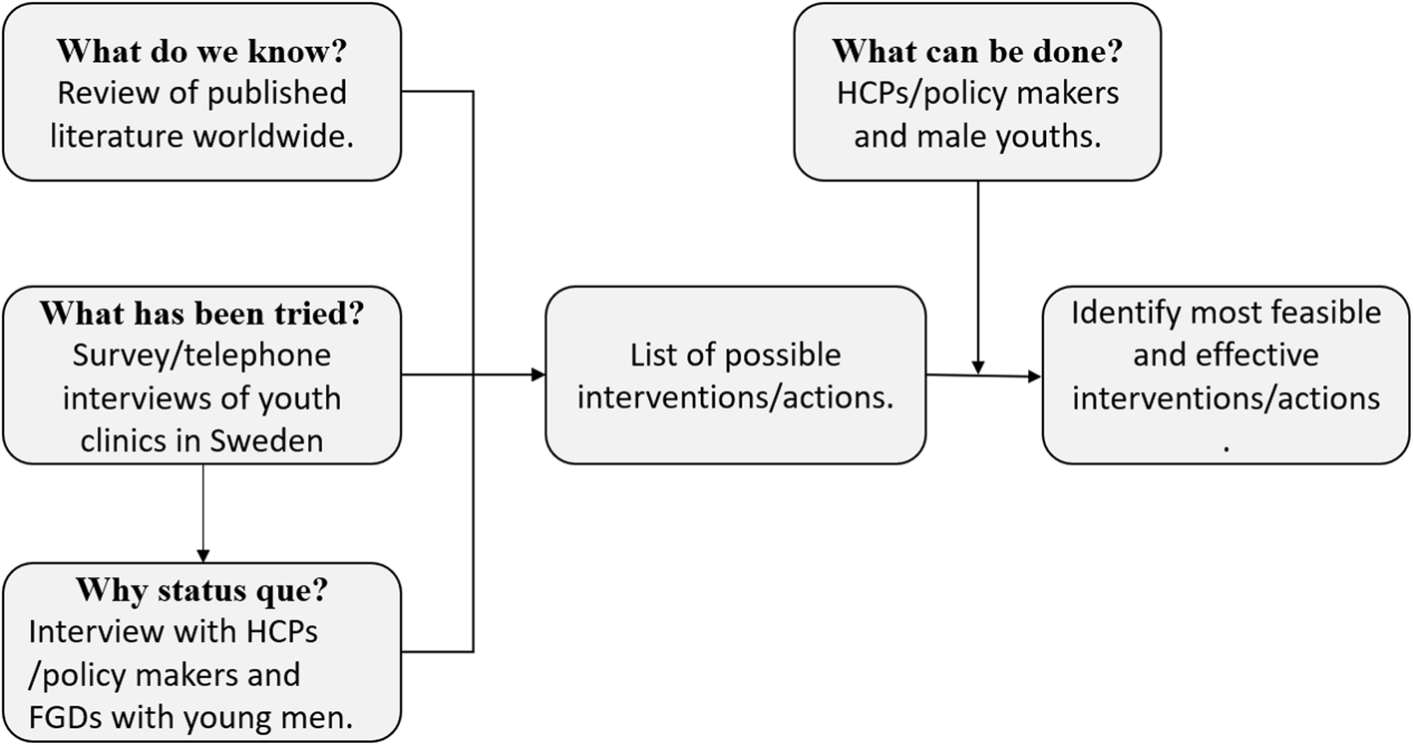
Study design and phases of the research project

This project will involve a mixed-methods design (case studies including individual reviews and focus group discussions (FGDs), systematic literature review, explorative survey, document review, and Q-sorting). The project comprises four phases corresponding to the four research questions. The phases are overlapping and build on each other’s. Phase 3 builds on phase 2 and phase 4 builds on the results of phase 1, 2 and 3 (Fig. 1).

### Data collection and analysis

In the *planning phase*, I have recruited an advisory committee (*n* = 17) with representatives from the Swedish Society for Youth Centres (FSUM); the Swedish Association for Sexuality Education (RFSU); and youth clinics in the largest regions of Sweden (Stockholm, Gothenburg, Malmö, and Uppsala beside Västernorrland and Västerbotten) including mangers (enhetschef), business developer (verksamhetsutvecklare) and HCPs. The advisory committee in its consultancy capacity will provide varied and complementary qualifications, experiences and skills that will contribute to achieving the aim of this research in a multidisciplinary approach. The brainstorming session at the start of the project and the continuous contact with the advisory committee will help to capture this interest groups’ opinions about the project including data collection (cases and participants’ selection and interview and focus group’s guides), interpretation of data, and dissemination activities. Besides providing insights about the project, the committee will have a consultancy role and oversee the process of the research providing feedback on preliminary findings. This will guarantee a continuous engagement of these stakeholders in the research process and will facilitate the dissemination and the translation of the findings into clinical practice and public health policies.

#### Phase 1: systematic review

The aim of this phase is to review and synthesize the literature on interventions aiming to increase boys and young men’s access to SRH services.

The review will be carried out using Arksey and O’Malley methods’ stages which include research question formulation, identifying relevant studies, study selection, data extraction, and summarizing and synthesizing the results [24]. Research question formulation will include a preparatory mapping, question wording and structure, search strategies and inclusion criteria. I will search the databases: PubMed, Cochrane, CINHAL, PsycINFO and Scopus with focus on empirical peer-reviewed studies published in English in the last twenty years about interventions and strategies to increase boys and young men’s access to SRH services in Europe. The systematic review protocol is registered in PROSPERO with ID number: CRD42024523589.

#### Phase 2: mapping initiatives

The aim of the second phase of this project is to explore the strategies that are used to attract boys and young men to youth clinics in Sweden.

##### Participants and recruitment

A contact list of all youth clinics in Sweden will be compiled. Managers or development officers will be contacted to complete the survey. There are approximately 250 youth clinics in Sweden. No sampling will be conducted; instead, all youth clinics in Sweden will be invited to participate.

##### Data collection

An exploratory survey will be distributed to all youth clinics in Sweden (250+) to investigate the strategies currently employed or previously attempted to engage boys and young men. The survey will inquire about the types of strategies utilized, if any, and their impact on the access of boys and young men to youth clinics. Short follow-up telephone interviews will be conducted with the clinics to gain further insights into these interventions as needed.

##### Data analysis

Quantitative data will be analysed descriptively, while qualitative data (from open-ended questions and the follow-up telephone interviews) will be analysed using qualitative content analysis.

#### Phase 3. case studies

The aim of this phase is to examine how individual and structural factors influencing the access of boys and young men to youth clinics in various regions of Sweden. Contextual elements such as healthcare organization, service availability, and regional sociodemographic structure may significantly impact access. To explore these factors, I will employ Yin RK’s multiple case study approach [25].

##### Participants and recruitment

With guidance from the advisory committee and phase 2 findings, two regions with diverse demographics and geography will be chosen. In these regions, the experiences, organization, and resources of youth clinics concerning boys’ and young men’s sexual and reproductive health (SRH) will be mapped based on phase 2 results. We will include three to four youth clinics from each region. HCPs, managers, and policymakers from these regions will be invited to participate in interviews (10 to 12 interviews per region). Focus group discussions (2 to 3 per region) will be conducted with boys and young men, both those who have and haven’t visited youth clinics. The recruitment strategy will be continually evaluated to ensure an understanding of each region’s context regarding boys’ and young men’s SRH.

##### Data collection

Thematic interview and focus group discussion guides will cover topics such as experiences of boys and young men in youth clinics, reasons for visiting or not visiting, and strategies to attract more visitors. A pilot interview/FGD will be conducted to refine the interview guides based on participant feedback and interview analysis. Participants for the first step, involving regional-level interviews with clinic managers and developers, will be identified through existing contacts, regional websites, and FSUM. Subsequently, healthcare providers at youth clinics will be selected based on discussions from the first step and publicly available information. Lastly, boys and young men in the region will be invited to focus group discussions through advertisements distributed via clinics, schools, and other venues. Schools will be contacted to ensure a diverse participant pool. Potential participants will be contacted via email and/or phone to explain the project’s purpose and seek consent for interviews/FGDs.

##### Data analysis

Recorded interviews/FGDs will be transcribed verbatim and analyzed using thematic analysis [26]. Each region will be analyzed separately before examining variations and commonalities between cases [25].

#### Phase 4: ordering strategies

The final phase of the project aims to identify effective and feasible strategies that help increasing boys and young men’s access to youth clinics in Sweden. Utilizing Q-sort (also known as Q-methodology), a mixed methodology approach, will enable a systematic exploration of variations and commonalities (shared meanings) in participants’ viewpoints [27]. This method overcomes the challenges inherent in quantitative research, where recruiting large sample sizes poses difficulties; with Q-sort, 40–60 participants are sufficient to discern specific viewpoints [28]. The Q-sort process includes five steps:

1. *Concourse development*: representing all statements regarding boys and young men’s access to youth clinics and strategies to increase their access to youth clinics. These statements will be derived from the literature review (phase one), the mapping of initiatives (phase two), the case studies (phase three) and inputs from key informants (the advisory committee).
2. *Item sampling (Q-Set)*: representing sample of statements from the concourse, organized *into sub-themes such as organizational changes in youth clinics*,* multi-sectoral approaches*, and outreach activities. A final set of statements, capturing diverse perspectives on boys and young men’s access to youth clinics, will be selected for ranking.
3. *Selection of participants (P-set)*: recruiting two participant groups with assistance from the advisory committee and phase two participants: 40–60 boys and young men, and 40–60 HCPs and policymakers. Participants will be invited with the support of FSUM (Swedish youth clinics’ association) and those involved in phase three of this research project.
4. *Q-sorting*: the participants will rank-order statements from the Q-set based on their perceptions of feasibility and effectiveness in enhancing boys and young men’s access to youth clinics. This task will be conducted online using Q-sortware (https://qsortware.net/). Additionally, participants will provide explanations for their sorting decisions through a series of open-ended questions.
5. *Q-analysis and factor interpretation*: conducting a bi-person factor analysis to identify shared perspectives, represented by factors. This analysis will facilitate the identification of strategies that potentially increase boys and young men’s access to youth clinics, as well as the exploration of variations and commonalities between the viewpoints of HCPs, policymakers, and boys and young men.

## Discussion

The World Health Organization and other global health actors have emphasized the importance of addressing the gap in SRH efforts by involving men and challenging harmful masculine norms [15]. Moreover, Sweden’s national SRHR strategy emphasizes the principle of health equality and equitable access to care: “women and men, girls and boys, must have the same conditions for good health and be offered care on equal terms” [29]. The Swedish Society for Youth Centers (FSUM) has also highlighted the need to enhance youth clinics’ capacity to address the SRH needs of boys and young men [6]. However, despite these calls to action, the Swedish national SRHR strategy lacks specific policy recommendations for improving SRH services for boys and young men [29]. Consequently, there remains a significant gap in both evidence and approaches aimed at engaging boys and young men in SRH services in Sweden.

Although the youth clinics in Sweden constitute a comprehensive example of differentiated youth friendly health services that have served the youths in Sweden since the 70s, the clinics struggle with reaching boys and young men until now [17, 18]. This paper presents a study protocol using a mixed method approach to identify effective strategies to attract boys and young men to youth clinics in Sweden with a view to improve their SRH and ultimately the enjoyment of rights among both young men and women. The methodological approach, involving systematic review, mapping exercise, case studies and Q-methodology, combines quantitative and qualitative methods in a way that better answers the research questions.

The project is a timely response to calls from the Swedish national strategy for SRHR [29], which identified young people as a priority group whose SRHR to be improved. The project will contribute new insights, inform policymakers, and facilitate the implementation of initiatives to enhance access to youth clinics for boys and young men. This is crucial for (1) devising gender-sensitive services and service delivery models to promote young men’s SRH; (2) informing future young men’s health policies; and (3) improving young men’s participation in SRH provision. Ultimately, this will lead to improvements in boys’ and young men’s SRH, fostering a culture of shared responsibilities and advancing gender equality.

However, achieving equitable health and rights still presents numerous challenges. Firstly, while this project aims to inform the health system’s response to increase the access of boys and young men to youth clinics, there remains a need for societal interventions adopting a gender-transformative approach. These interventions are crucial in altering the perception of youth clinics as exclusively for women or for STI testing.

Secondly, securing support and resources to implement and institutionalize the identified interventions poses a significant challenge. Resistance from both HCPs and institutions is to be anticipated. Competing priorities and limited resources within healthcare systems could hinder the implementation of comprehensive and effective interventions.

## Data Availability

No datasets were generated or analysed during the current study.

## Abbreviations

FGDs: Focus group discussions
FSUM: Swedish youth clinics’ association
HCPs: Healthcare providers
HIV: Human immunodeficiency virus
STIs: Sexually transmitted infections
SRH: Sexual and reproductive health
SRHR: Sexual and reproductive health and rights
